# Modulation instability in nonlinear media with sine-oscillatory nonlocal response function and pure quartic diffraction

**DOI:** 10.1038/s41598-024-59722-z

**Published:** 2024-04-18

**Authors:** Yuwen Yang, Ming Shen

**Affiliations:** Institute for Quantum Science and Technology, Department of Physics, Shanghai University, Shanghai, 200444 China

**Keywords:** Nonlinear optics, Solitons

## Abstract

Modulation instability of one-dimensional plane wave is demonstrated in nonlinear Kerr media with sine-oscillatory nonlocal response function and pure quartic diffraction. The growth rate of modulation instability, which depends on the degree of nonlocality, coefficient of quartic diffraction, type of the nonlinearity and the power of plane wave, is analytically obtained with linear-stability analysis. Different from other nonlocal response functions, the maximum of the growth rate in media with sine-oscillatory nonlocal response function occurs always at a particular wave number. Theoretical results of modulation instability are confirmed numerically with split-step Fourier transform. Modulation instability can be controlled flexibly by adjusting the degree of nonlocality and quartic diffraction.

## Introduction

Modulation instability (MI) refers to spontaneous growth of modulation or perturbations in the amplitude or phase of a wave propagating through a nonlinear media, which usually occurs when the delicate balance between dispersion and nonlinearity is disrupted, leading to the self-induced generation of sidebands in the waveform^1^. MI is a fascinating phenomenon in various physical systems^2^. During the past two decades, modulation instability (MI) has been widely studied in nonlocal nonlinear media^2–9^. Nonlocality means that the response of the media at a particular point is not determined solely by the wave intensity at that point(as in local media), but also depends on the wave intensity in its vicinity^2^. It has been shown that properties of MI, such as maximum and bandwidth of the growth rate, can be greatly affected by spatial nonlocality^3,4^. Transverse instability and dynamics of bright soliton stripes in two-dimensional nonlocal nonlinear media were investigated using multi-scale perturbation method^10^. In the recent years, MI has also been investigated in the presence of competing nonlocal nonlinearities^11–16^.

Generally, characteristics of nonlocal MI are related to the specific form of nonlocal response function, e.g., Gaussian, exponential, and rectangular nonlocal response functions^3,4^ have been used to study MI in nonlocal media. These response functions are always positive definite. Recently, MI has been also demonstrated in nonlocal media with sine-oscillatory response function^17–19^. This kind of nonlocal response function can be proposed in quadratic nonlinear media^20,21^ and nematic liquid crystals with negative dielectric anisotropy^22,23^. It has been shown that nonlocal media with sine-oscillatory response function provides new physical mechanisms on chaotic dynamics^24,25^, solitons induced by boundary confinement^26–28^, and novel solitons states^29–31^.

In the recent years, the effects of higher-order, in particular, quartic (fourth-order) dispersion/diffraction on MI have attracted much interest. In nonlinear optical fibre, quartic group velocity dispersion introduces novel features of MI^32–38^. In spatial domain, quartic diffraction is an unique property of photonic crystals with subdiffractive effect^39,40^, periodic structure consisting of both positive and negative index materials^41,42^, and microstructure of cavity^43–47^. In local nonlinear media with quartic diffraction, Zhang et. al. have studied transverse instability^48^ and spatiotemporal instability^49^ with linear-stability analysis. Recently, MI is also investigated in nonlocal nonlinear media with competing cubic and quintic nonlocal nonlinearities and quartic diffraction^50^. In the regime of weak nonlocality, MI has been studied with quartic dispersion^51,52^. However, previous works^50–52^ have not considered nonlocal media with sine-oscillatory nonlocal response function when quartic dispersion/diffraction is taken into account.

In this paper, we study analytically and numerically MI of one-dimensional plane wave in nonlinear Kerr media with sine-oscillatory nonlocal response function and pure quartic diffraction. Using linear-stability analysis, the growth rate of MI is obtained which shows that the degree of nonlocality, coefficient of quartic diffraction, type of the nonlinearity and the power of plane wave have deep impacts on maximum and bandwidth of MI spectra. The maximum of the growth rate occurs always at a particular wave number. We also demonstrate properties of MI with split-step Fourier transform. Nonlocality and quartic diffraction can suppress or promote MI flexibly.

**Figure 1:**
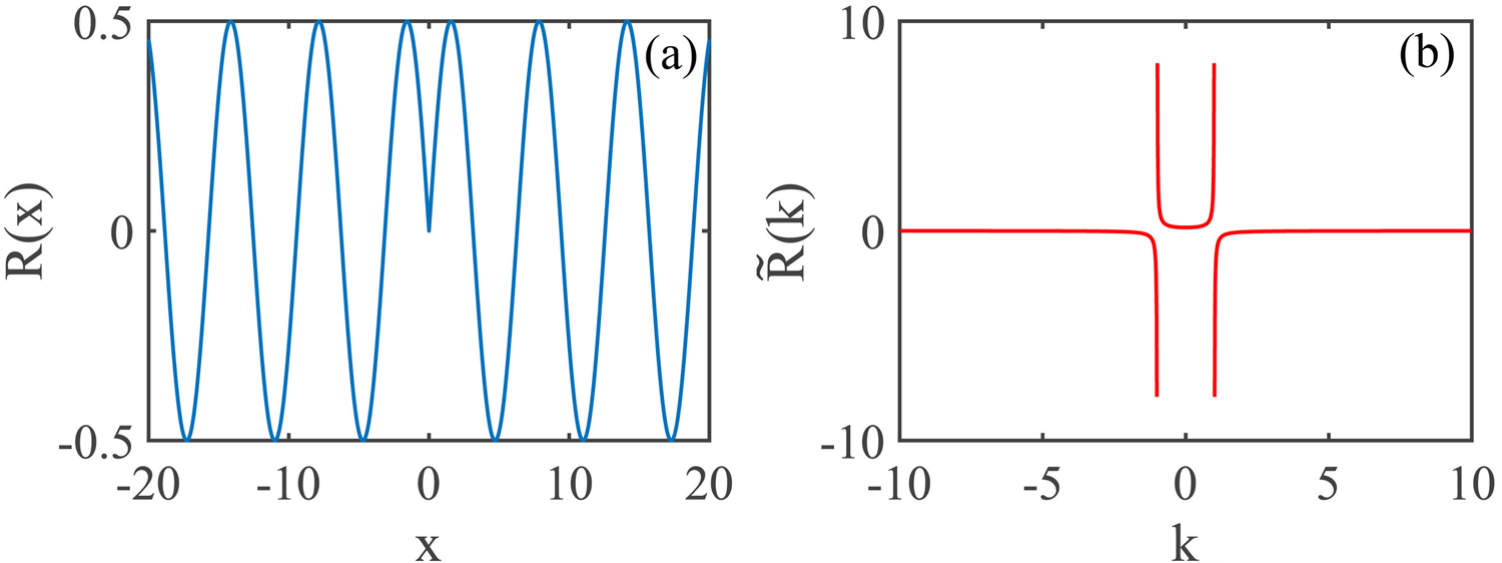
The sine-oscillatory nonlocal response function (**a**) and its Fourier transform (**b**). The degree of nonlocality is $\sigma =1$.

## Method

### Model and basic equations

Considering an one-dimensional optical beam propagating in a nonlocal nonlinear media with pure quartic diffraction, the dynamics of such beam can be described by the following normalized nonlocal nonlinear Schrödinger equation^50^

1 $$\begin{aligned} i\frac{\partial \psi }{\partial z}+\beta _{4}\frac{\partial ^4 \psi }{\partial x^4}+s\psi \int R({x-{x}'})|\psi (x',z)|^2d^2{{x}'}=0, \end{aligned}$$

where the variables *x* and *z* are dimensionless spatial coordinates. The parameter $\beta _{4}$ corresponds to quartic diffraction coefficient of the beam ($\beta _4>0$ and $\beta _4<0$ represent anomalous and normal diffractions^53,54^, respectively), and $s=1$ ($s=-1$) represents a focusing (defocusing) nonlocal nonlinearity. *R*(*x*) is nonlocal response function which has several different representations, such as the Gaussian function^55^, rectangular function^2–4^. In this paper, we assume the response function is in the following sine-oscillation form^22,23^

2 $$\begin{aligned} R(x)=\frac{1}{2\sigma }\sin \left( \frac{\left| x \right| }{\sigma }\right) , \end{aligned}$$

with the Fourier transform of the nonlocal response function $\tilde{R}(x)$ is represented as

3 $$\begin{aligned} \tilde{R}(k)= \frac{1}{2\pi }\int R(x)\exp {(-ikx)}d{x}=\frac{1}{2\pi (1-\sigma ^{2}k^{2})}. \end{aligned}$$

The sine-oscillatory nonlocal response function and its Fourier transform are shown in Fig. 1a,b, respectively.

**Figure 2:**
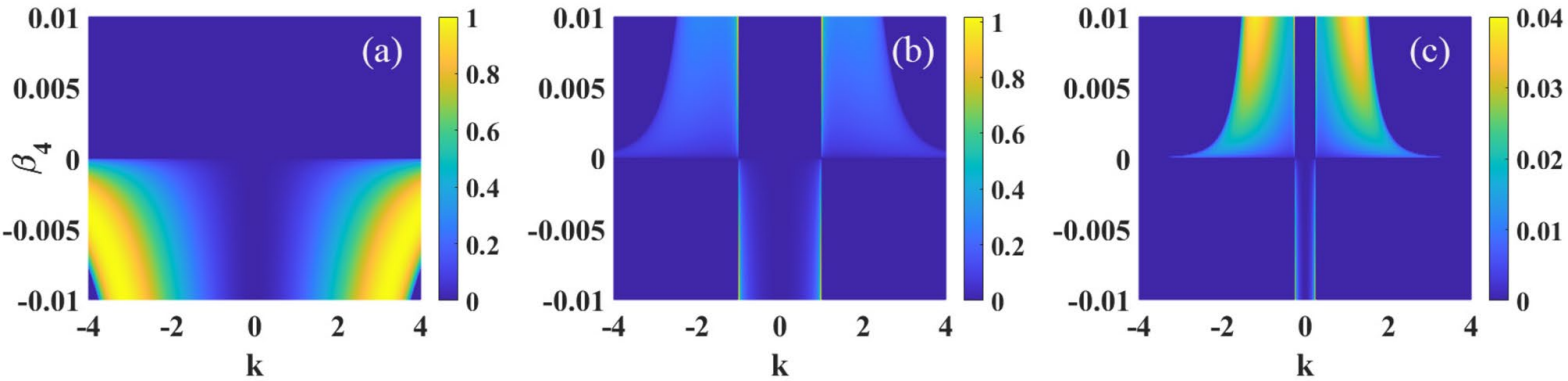
The MI gain spectra versus the wave number *k* and quartic diffraction parameter $\beta _{4}$, for $s=1$ and $P_{0}=1$. The other parameter are: (**a**) $\sigma =0$, (**b**) $\sigma =1$ and (**c**) $\sigma =4$.

### Linear-stability analysis

In general, the plane wave solution of Eq. (1) can be written as^17–19^

4 $$\begin{aligned} \psi =\sqrt{P_0}\exp {(i2\pi \tilde{R}(0)sP_0 z)}, \end{aligned}$$

here $P_0$ is optical intensity of uniform plane wave.

Then, we introduce a random perturbation *a*(*x*, *z*) to the plane wave solution

5 $$\begin{aligned} \psi (x,z)=[\sqrt{P_0}+a(x,z)]\exp {(i2\pi \tilde{R}(0)sP_0 z)} \end{aligned}$$

with ${\left| a \right| } ^2\ll P_0$. Substituting Eq. (5) into Eq. (1) and linearizing around the unperturbed solution, we can obtain

6 $$\begin{aligned} i\frac{\partial a}{\partial z}+\beta _{4}\frac{\partial ^4 a}{\partial x^4}+2sP_0\int R({x}-{x'})\text {Re} [a(x',z)]d^2{x'}=0. \end{aligned}$$

Decomposition the perturbation into the complex form of $a=u+iv$ with *u* and *v* are real and the imaginary parts, respectively, then we can obtain the following two coupled equations

7 $$\begin{aligned}&\frac{\partial u}{\partial z}+ \beta _{4}\frac{\partial ^4v}{\partial x^4}=0, \end{aligned}$$

8 $$\frac{{\partial v}}{{\partial z}} - \beta _{4} \frac{{\partial ^{4} u}}{{\partial x^{4} }} - 2sP_{0} \int R (x - x^{\prime } )u(x^{\prime } ,z)d^{2} x^{\prime } = 0.$$

Equations (7) and (8)

9 $$\begin{aligned}&\frac{\partial \tilde{u}}{\partial z}+\beta _{4}k^4\tilde{v}=0, \end{aligned}$$

10 $$\begin{aligned}&\frac{\partial \tilde{v}}{\partial z}-\beta _{4}k^4\tilde{u}-4\pi s P_0 \tilde{R}(k)\tilde{u}=0, \end{aligned}$$

here

11 $$\begin{aligned} \tilde{u}(k,z)= \int u(x,z)\exp {(ikx)}dx, \end{aligned}$$

12 $$\begin{aligned} \tilde{v}(k,z)= \int v(x,z)\exp {(ikx)}dx, \end{aligned}$$

are Fourier transform of *u*(*x*, *z*) and *v*(*x*, *z*), respectively.

**Figure 3:**
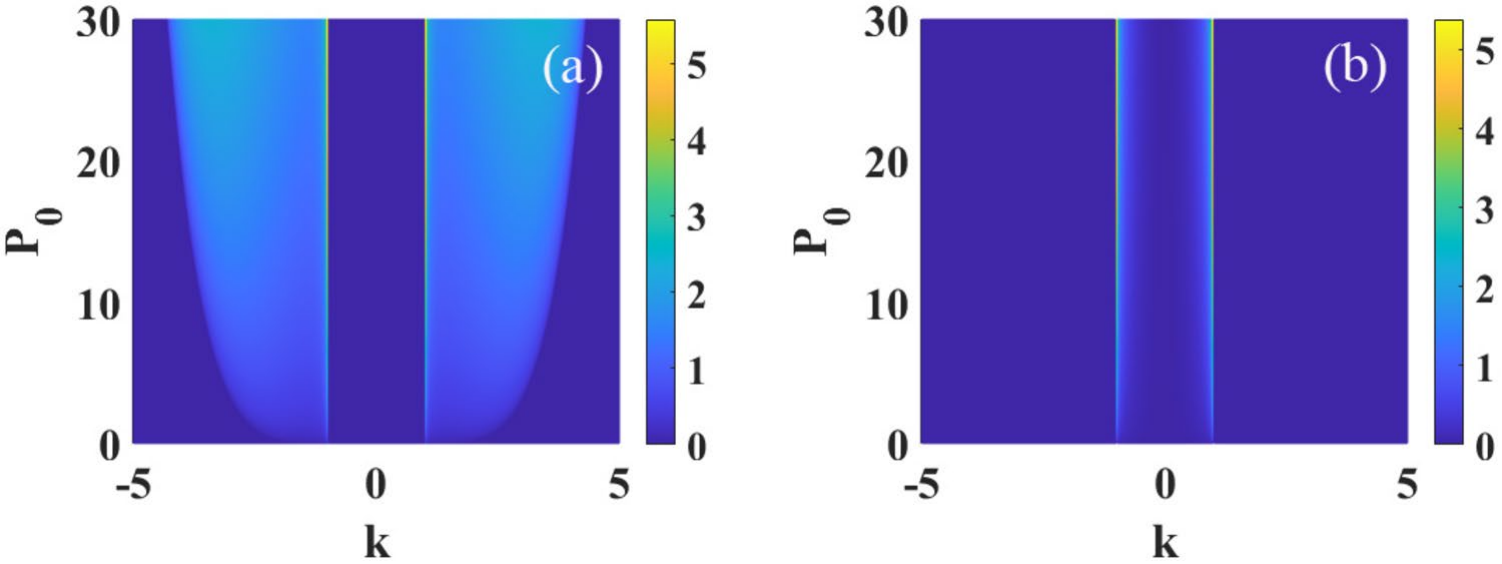
The MI gain spectra versus the wave number *k* and optical intensity $P_0$, for $s=1$ and $\sigma =1$. The other parameter are: (**a**) $\beta _{4}=0.01$ and (**b**) $\beta _{4}=-0.01$.

Considering the derivatives of Eqs. (9) and (10) with respective to coordinate *z*, we can obtain the following ordinary differential equations in the *k* space

13 $$\begin{aligned}&\frac{\partial ^2u}{\partial z^2}+\beta _{4}k^4[(\beta _{4}k^4+4\pi s P_0 \tilde{R}(x))]\tilde{u}=0, \end{aligned}$$

14 $$\begin{aligned}&\frac{\partial ^2v}{\partial z^2}+\beta _{4}k^4[(\beta _{4}k^4+4\pi s P_0 \tilde{R}(x))]\tilde{v}=0. \end{aligned}$$

**Figure 4:**
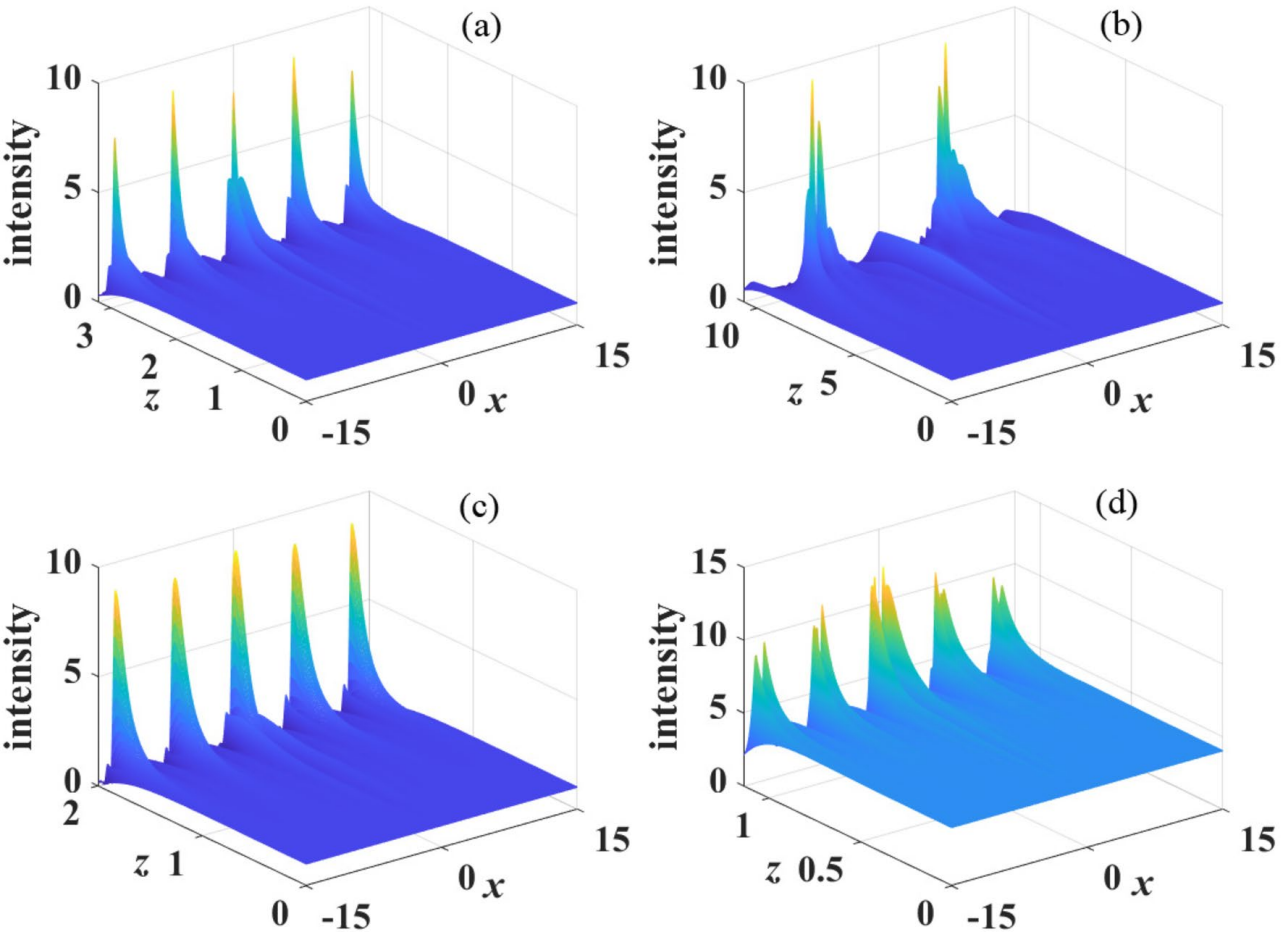
The propagation of perturbed plane wave in kerr media with focusing nonlinearity($s=1$). The parameter are: (**a**) $\sigma =1$, $\beta _{4}=0.01$, $P_0=1$; (**b**) $\sigma =2$, $\beta _{4}=0.01$, $P_0=1$; (**c**) $\sigma =1$, $\beta _{4}=0.05$, $P_0=1$ and (**d**) $\sigma =1$, $\beta _{4}=0.01$, $P_0=2$.

By solving Eqs. (13) and (14), the solution of random perturbation

15 $$\begin{aligned} \tilde{a}(k,z)=u+iv=c_1\exp {(\lambda z)}+c_2\exp {(-\lambda z)}, \end{aligned}$$

is obtained with $c_1$ and $c_2$ are arbitrary constants, and the eigenvalue $\lambda$ is given by

16 $$\begin{aligned} \lambda ^2=-\beta _{4}k^4[\beta _{4}k^4+4\pi s P_0\tilde{R}(k)]. \end{aligned}$$

It is obvious that no MI exists when $\lambda ^{2} < 0$ and the plane wave is stable. On the contrary, for $\lambda ^{2}>0$, the perturbation grows exponentially during propagation. The growth rate defined by $g(k) = \left| \text {Re}\left\{ \lambda \right\} \right|$ is represented as

17 $$\begin{aligned} g(k)= & {} k^2*\text {Re}\left\{ \sqrt{-\beta _{4}(4\pi s P_0\tilde{R}(k)+\beta _{4}k^4)}\right\} \nonumber \\= & {} k^2*\text {Re}\left\{ \sqrt{-\beta _{4}(\frac{2sP_0}{1-\sigma ^{2}k^2 } +\beta _{4}k^4)}\right\} , \end{aligned}$$

which indicates that MI exists only when $2sP_0/(1-\sigma ^{2}k^2) +\beta _{4}k^4<0$. In the limit of local nonlinearity, i.e., $R(x) =\delta (x)$ and $\sigma = 0$, the growth rate is

18 $$\begin{aligned} g(k)=k^2*\text {Re}\left\{ \sqrt{-\beta _{4}(4\pi s P_0+\beta _{4}k^4)}\right\} . \end{aligned}$$

**Figure 5:**
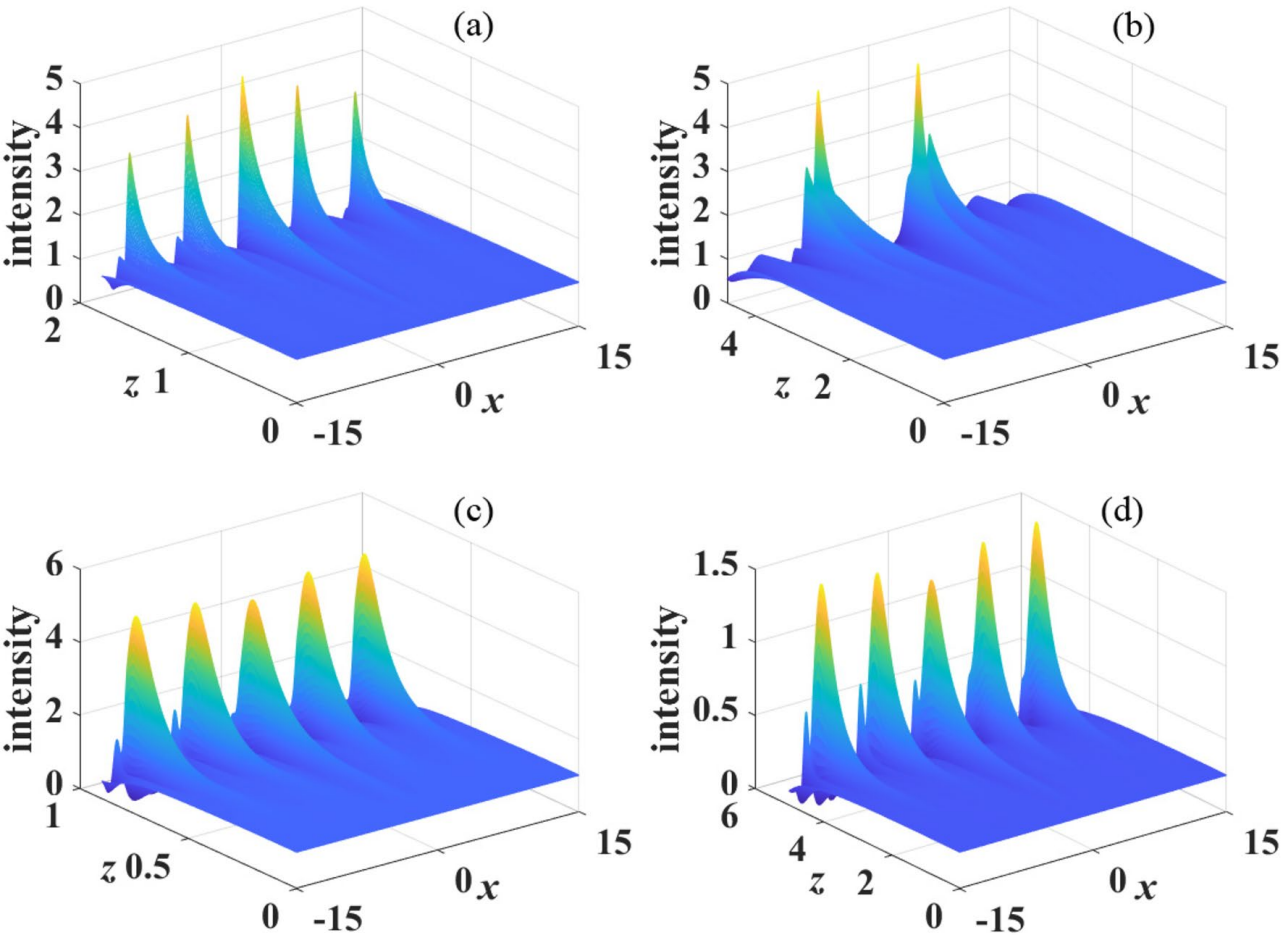
The propagation of perturbed plane wave in kerr media with focusing nonlinearity($s=1$). The parameter are: (**a**) $\sigma =1$, $\beta _{4}=-0.05$, $P_0=1$; (**b**) $\sigma =2$, $\beta _{4}=-0.05$, $P_0=1$; (**c**) $\sigma =1$, $\beta _{4}=-0.5$, $P_0=1$ and (**d**) $\sigma =1$, $\beta _{4}=-0.05$, $P_0=0.5$.

## Results

### MI when s = 1

Firstly, we focus on MI in self-focusing nonlocal Kerr media with $s=1$. We display in Fig. 2 the MI gain spectra versus the wave number *k* and quartic diffraction coefficient $\beta _{4}$. In the limit of local nonlinearity ($\sigma =0$), as shown in Fig. 2a, there are two symmetric sidebands when $\beta _{4}<0$ and the bandwidth decreases when $\beta _{4}$ decreases. However, MI disappears when $\beta _{4}>0$. When the degree of nonlocality is weak $\sigma =1$, as shown in Fig. 2b, the sidebands appear regardless of the quartic diffraction is normal or anomalous. When $\beta _{4}<0$, the maximum of growth rate increases with the decrease of $\beta _{4}$ while the bandwidth remains constant. On the contrary with $\beta _4>0$, when $\beta _4$ increases, the bandwidth will decrease, while the maximum of the growth rate will increase. Thus MI can be suppressed with smaller $|\beta _4|$. As shown in Fig. 2c, when the degree of nonlocality is $\sigma =4$, we can find that both the maximum and the bandwidth of the growth rate decrease, which indicates that MI can be effectively suppressed with strong nonlocality.

**Figure 6:**
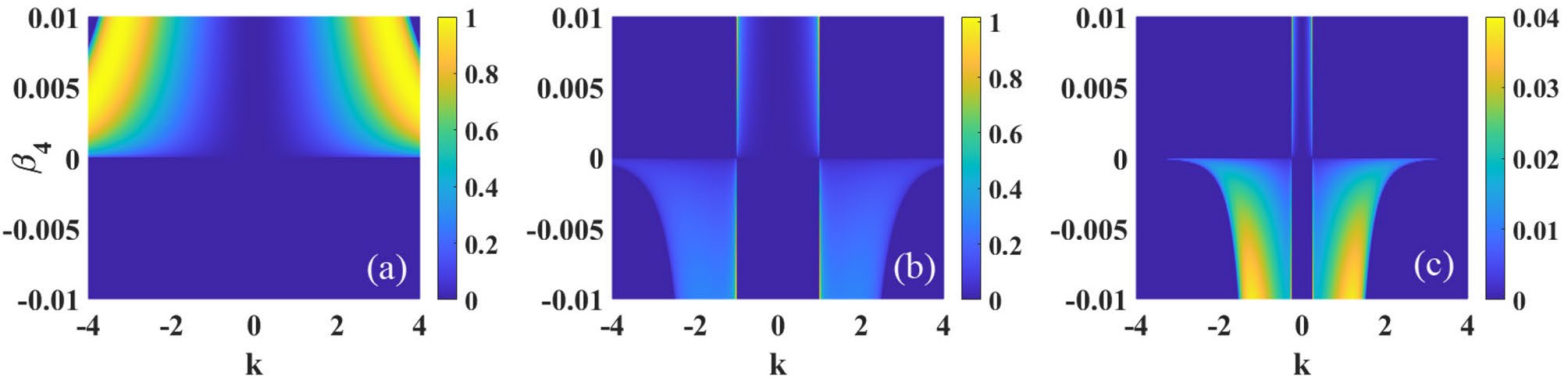
The MI gain spectra versus the wave number *k* and quartic diffraction parameter $\beta _{4}$, for $s=-1$ and $P_{0}=1$. The other parameter are: (**a**) $\sigma =0$, (**b**) $\sigma =1$ and (**c**) $\sigma =4$.

Figure 3 illustrates the influences of $P_{0}$ on MI. In the case of $\beta _{4}>0$, bandwidth and maximum of growth rate increase with the increase of $P_{0}$, as shown in Fig. 3a. However, in the case of $\beta _{4}<0$, as shown in Fig. 3b, the maximum of the growth rate increases while the bandwidth remains constant when $P_{0}$ increases. Thus, the increase of optical intensity $P_0$ promotes MI regardless the quartic diffraction is normal or anomalous. Furthermore, different from other nonlocal response functions^50^, we also find that the maximum of the growth rate occurs always at the particular wave number $\left| k \right| =1/\sigma$, as shown in Figs. 2 and 3.

**Figure 7:**
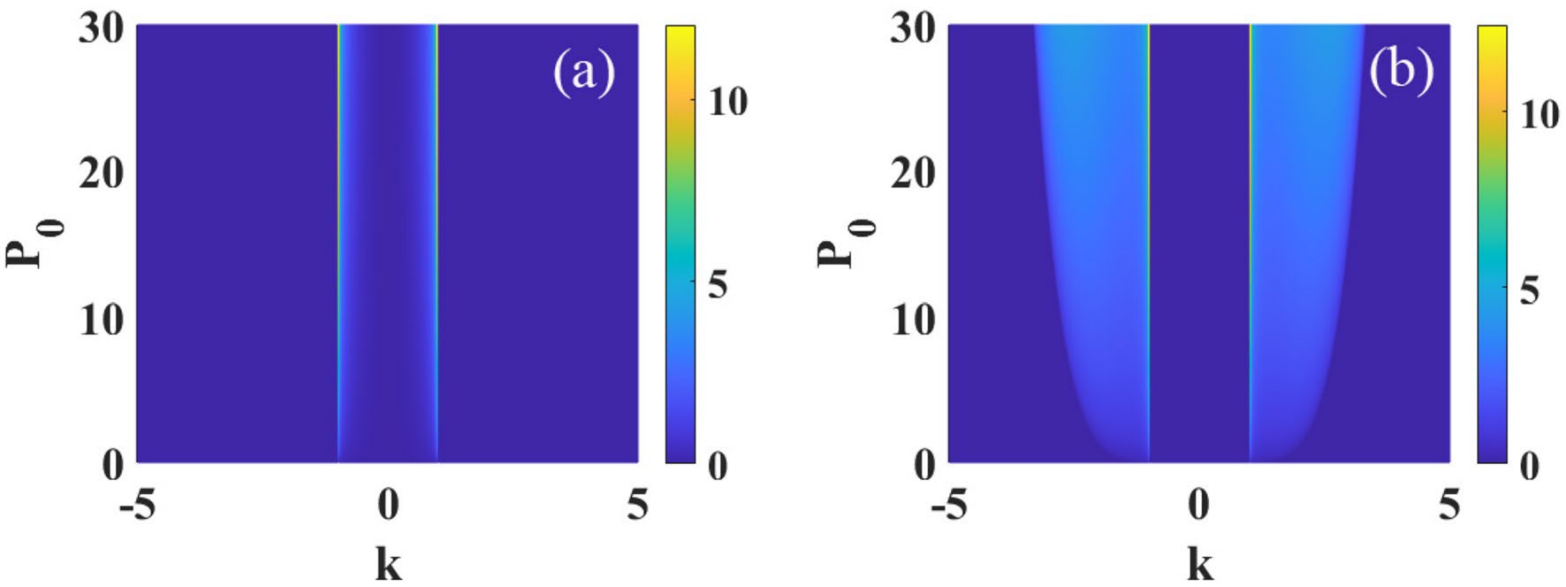
The MI gain spectra versus the wave number *k* and optical intensity $P_0$, for $s=-1$ and $\sigma =1$. The other parameter are: (**a**) $\beta _{4}=0.05$ and (**b**) $\beta _{4}=-0.05$.

To demonstrate the MI obtained by linear-stability analysis in self-focusing Kerr media with a sine-oscillatory nonlocal response function, we perform numerical simulations of Eq. (1) by using split-step Fourier method. A plane wave with a small period perturbation is used as the initial input

19 $$\begin{aligned} \psi (x,z=0)=\sqrt{P_0}+\varepsilon \cos (kx), \end{aligned}$$

with amplitude $\varepsilon =10^{-4}$ and the wave number *k* (corresponds to the maximum of the growth rate) of the perturbation.

When $\beta _{4}>0$, we show in Fig. 4 the propagation dynamics of the perturbed plane wave in nonlocal self-focusing media with different parameters. We can see that the perturbation grows obviously at propagation distance $z=3$ with $\beta _{4}=0.01$, $P_{0}=1$ and $\sigma =1$, as displayed in Fig. 4a. When the degree of nonlocality increases ($\sigma =2$), as shown in Fig. 4b, MI is suppressed significantly. Almost no MI exist at $z=3$, and perturbation grows visibly at $z=10$. This result conforms to the conclusion of Fig. 2 that MI can be effectively suppressed with strong nonlocality. Figure 4c,d also confirm the conclusions that MI can be promoted by increasing $\beta _4$ and $P_0$, which have been illustrated in Figs. 2 and 3.

**Figure 8:**
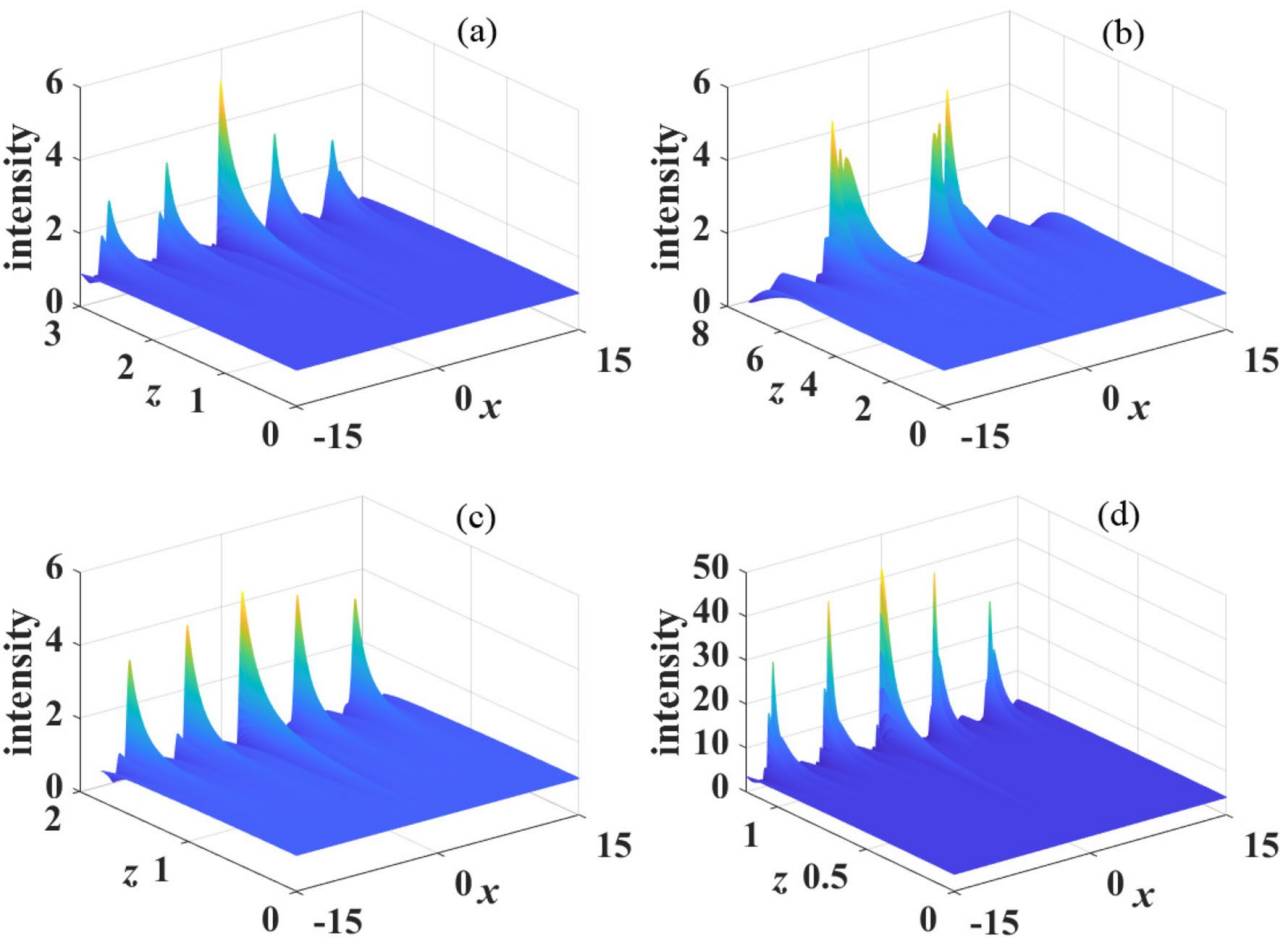
The propagation of perturbed plane wave in kerr media with defocusing nonlinearity($s=-1$). The parameter are: (**a**) $\sigma =1$, $\beta _{4}=0.01$, $P_0=1$; (**b**) $\sigma =2$, $\beta _{4}=0.01$, $P_0=1$; (**c**) $\sigma =1$, $\beta _{4}=0.05$, $P_0=1$ and (**d**) $\sigma =1$, $\beta _{4}=0.01$, $P_0=2$.

Numerical simulations of the propagation of perturbed plane waves are displayed in Fig. 5 in the case of $\beta _{4}<0$. Compare Fig. 5a with Fig. 5b, similar with $\beta _{4}>0$, strong nonlocality also suppress MI. It is also demonstrated that MI is enhanced with the decrease of $\beta _4$ and weakened when $P_0$ decrease, as shown in Fig. 5c,d. These numerical simulations are in completely agreement with the analytical results obtained by linear-stability analysis.

### MI when

$$s=-1$$

Subsequently, we study the MI in nonlocal Kerr media with self-defocusing nonlinearity ($s=-1$). It is well known that MI in nonlocal self-defocusing media with second-order diffraction sensitively depends on the type of nonlocal response function^3^, whereas the introduction of fourth-order diffraction makes it possible for MI to occur in nonlinear media with arbitrary form of nonlocal response functions. Also standard diffraction is always positive (normal)^18^, on the contrary, quartic diffraction can be either positive or negative. Similarly, we display the gain spectra of MI with different parameters in Fig. 6. In contrast to the case of self-focusing nonlinearity, in the limit of local nonlinearity ($\sigma =0$), as shown in Fig. 6a, the sidebands of MI appear in the region $\beta _{4}>0$ and disappear in the region $\beta _{4}<0$. In nonlocal case, as shown in Fig. 6b,c, the sidebands appear for arbitrary quartic diffraction coefficients, and the maximum of growth rate increases when the absolute value of the quartic diffraction coefficients increases. The bandwidths keep invariant for anomalous diffraction ($\beta _4>0$) and decrease when $\beta _4$ decrease for normal diffraction ($\beta _4<0$). Moreover, when the degree of nonlocality increases, both the maximum of the growth rate and the bandwidth of MI spectra decrease. This suggests that the conclusion MI is eliminated by strong nonlocality can also be easily obtained.

**Figure 9:**
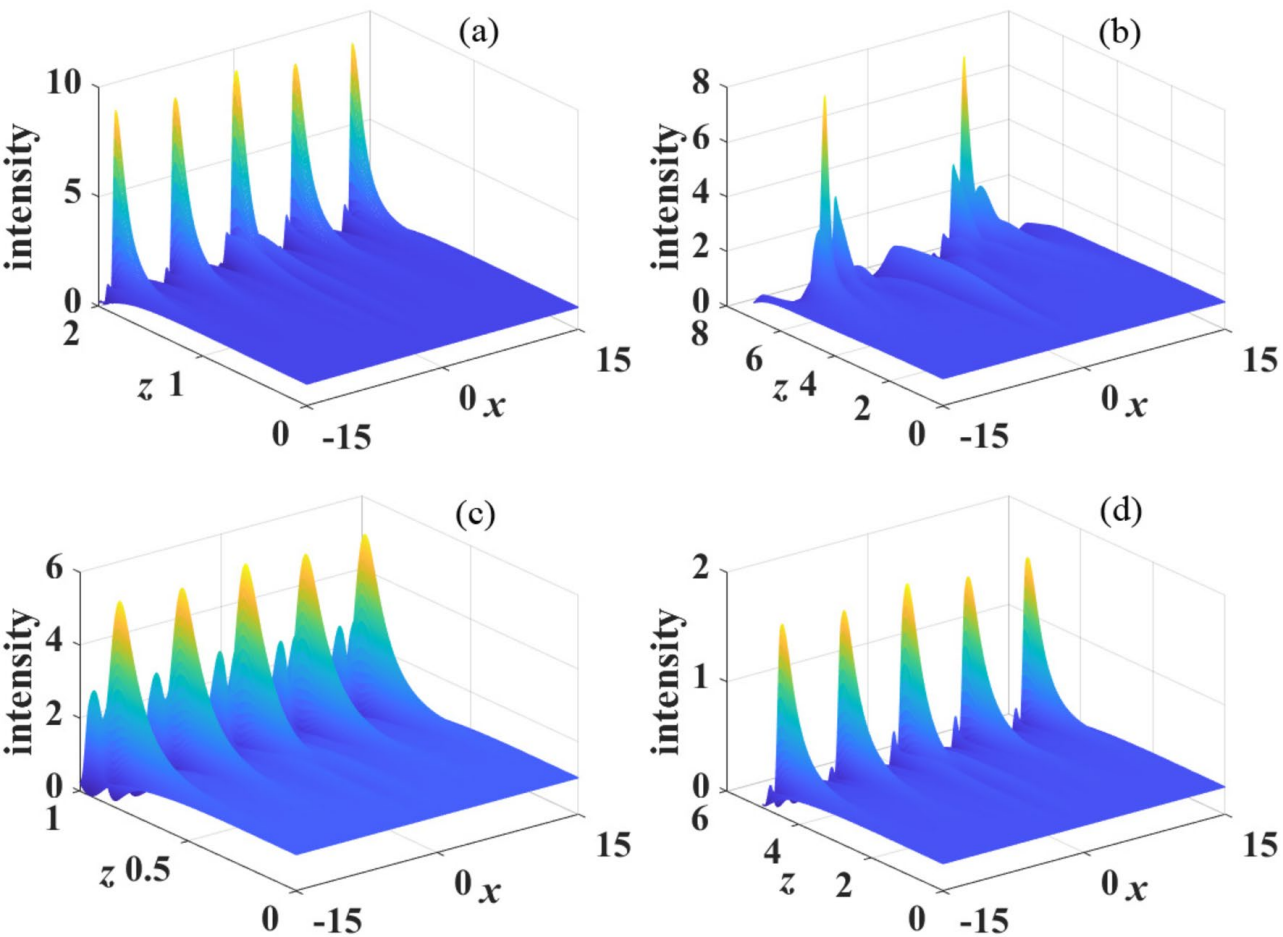
The propagation of perturbed plane wave in kerr media with defocusing nonlinearity($s=-1$). The parameter are: (**a**) $\sigma =1$, $\beta _{4}=-0.05$, $P_0=1$; (**b**) $\sigma =2$, $\beta _{4}=-0.05$, $P_0=1$; (**c**) $\sigma =1$, $\beta _{4}=-0.5$, $P_0=1$ and (**d**) $\sigma =1$, $\beta _{4}=-0.05$, $P_0=0.5$.

Similarly, the impact of power $P_{0}$ on the spectra of MI in a self-defocusing media are displayed in Fig. 7. The maximum of growth rate always increase with the increase of $P_{0}$ for both normal and anomalous quartic diffraction. The bandwidth remains constant for $\beta _{4}>0$ (Fig. 7a), whereas, as shown in Fig. 7b, in the region $\beta _{4}<0$, the bandwidth increases when $P_{0}$ increases. These results are opposite to the case of $s=1$. We also find that the wave number $\left| k \right| =1/\sigma$ has the maximum of the growth rate.

Numerical simulations of the propagation of perturbed plane wave (Eq. 19) are demonstrated in Figs. 8 and 9. Obviously, as shown in Figs. 8a,b and 9a,b, in the region $\beta _{4}>0$ and $\beta _{4}<0$, strong nonlocality still suppresses MI effectively. Moreover, for $\beta _{4}>0$, MI is weakened with the increase of $\beta _{4}$ and the decrease of $P_0$, as shown in Fig. 8c,d. However, for $\beta _{4}<0$, MI is weakened with the decrease of $\beta _{4}$ and $P_0$, as shown in Fig. 9c,d. These numerical results are also consistent with the analytical results obtained by linear-stability analysis (Figs. 6 and 7).

## Conclusions

In conclusion, we have investigated MI of one-dimensional plane wave in nonlinear Kerr media with sine-oscillatory nonlocal response functions and pure quartic diffraction. The growth rate of MI was analytically obtained with linear-stability analysis and confirmed numerically with split-step Fourier transform. MI are sensitive to the degree of nonlocality, coefficient of quartic diffraction, type of the nonlinearity as well as the power of plane wave. The maximum of the growth rate occurs always at particular wave number $|k|=1/\sigma$. Analytical and numerical results indicate that MI can be suppressed with the help of nonlocality and quartic diffraction.

## Data Availability

The datasets used and/or analysed during the current study available from the corresponding author on reasonable request.
